# Supplementary material for: Hybrid computational modeling demonstrates the utility of simulating complex cellular networks in type 1 diabetes
Source: PLoS Comput Biol. 2021 Sep 27;17(9):e1009413. doi: 10.1371/journal.pcbi.1009413 (PMC8496846; doi:10.1371/journal.pcbi.1009413)
Supplement: S1 Table — (DOCX) [file pcbi.1009413.s001.docx]

**S1 Table**. Agent types and shapes in agent-based modeling (ABM)

| **Agent type.** | **Agent shape** | **Description** |
| --- | --- | --- |
| Dendritic cells | 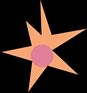 | Process antigens and present them to naïve CD8^+^T cells |
| Antigens | 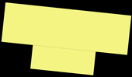 | Molecules released by β cells |
| Naïve CD8^+^T cells | 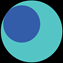 | Agents that respond to dendritic cells expressing antigens |
| Activated CD8^+^T cells | 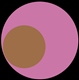 | Naïve CD8^+^T cells become activated |
| Cytotoxic CD8^+^T cells | 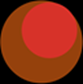 | Agents that damage β cells |
| Pancreatic β cells | 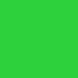 | Agents reside most likely in the center of islets |
| Pancreatic α cells | 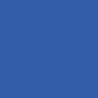 | Agents mostly reside in the periphery of islets |
| Pancreatic γ cells | 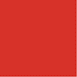 | Agents mostly reside in the periphery of islets |
