# Supplementary material for: Hybrid computational modeling demonstrates the utility of simulating complex cellular networks in type 1 diabetes
Source: PLoS Comput Biol. 2021 Sep 27;17(9):e1009413. doi: 10.1371/journal.pcbi.1009413 (PMC8496846; doi:10.1371/journal.pcbi.1009413)
Supplement: S2 Table — (DOCX) [file pcbi.1009413.s002.docx]

**S2 Table.** Simulation initialization and agent set up in ABM environment

| WORLD (ABM environment)  The world is a 201-unit x 201-unit square. |
| --- |
| Initialization (The initialization is set up to represent normal conditions)   1. A 201-unit x 201-unit square is created. The entire space is divided into three regions: pancreatic lymphatics, pancreatic islets, and circulation. 2. The pancreatic lymphatic system is located in the region where pancreatic lymph nodes (PLNs) are. For simplicity, the width of this region is set equal to the diameter of a pancreatic lymph node. 3. PLNs are created as “patches” to model locations of naïve CD8^+^T cells in the region of pancreatic lymphatics. They are structural agents and are used to set up the background for cell interactions and movements in PLNs. For simplification, the shape of each PLN was set to circle. The algorithm for setting up a PLN is as follows:   procedure: set up PLNs  for i_th_ PLN  define the center of i_th_ PLN (x, y)  check distance between i_th_ PLN and (i-1)_th_ PLN  if distance < diameter of PLN  define another center (x, y)  else  fill the circular space, where (x, y) is the center  end if  end for  end   1. Circulation regions are also structural agents, where circulating dendritic cells are created in this region when they were recruited during a T1D progression. 2. In the pancreas, islets can form a cluster, or randomly distributed in the pancreas. Centers of cluster were randomly selected in the pancreatic islet region. In each cluster, pancreatic islets randomly filled the space. The algorithm for setting up pancreatic clusters is given as follows:   procedure: set up islet clusters  for i_th_ islet cluster  define the center of i_th_ islet cluster (x, y)  check distance between i_th_ islet cluster and (i-1)_th_ islet cluster  if distance < diameter of islet cluster  define another center (x, y)  else  fill the circular space with individual islets  end if  end for  end procedure   1. Individual islets are randomly distributed in the remaining space of the pancreas. The shape of the pancreatic islet was simplified to a circle. Three types of cells are included in the pancreatic islets. They are pancreatic β cells, pancreatic α cells, and pancreatic γ cells. Pancreatic β cells are in the center area of a pancreatic islet [1], pancreatic α and γ cells are in the periphery area of a pancreatic islet [1]. They were created in different colors to distinguish the cell type. “Patches” representing pancreatic islets are used to model the site of T1D occurrence. 2. Circulating naïve CD8^+^T cells are created in PLNs. The initial number of circulating naïve CD4 T cells follows a normal distribution $\mathcal{\sim N}\left( \boldsymbol{15000,900} \right)$, which is comparable to the number of naïve CD8^+^T cells in experiments based on the model size (the model reflects approximately 1/100 of actual size) [2]. Naïve CD8^+^T cells are shown in blue circles in PLNs. 3. Resident dendritic cells are randomly distributed in the region of pancreatic islets. Based on the experimental finding [3], 7 dendritic cells are created in each pancreatic islet. Dendritic cells are shown by yellow “flower” shape agents in the region of pancreatic islets. |

**References**

1. Kim A, Miller K, Jo J, Kilimnik G, Wojcik P, Hara M. Islet architecture: a comparative study. Islets. 2009;1(2):129-36.

2. den Braber I, Mugwagwa T, Vrisekoop N, Westera L, Mögling R, de Boer AB, et al. Maintenance of peripheral naive T cells is sustained by thymus output in mice but not humans. Immunity. 2012;36(2):288-97.

3. Calderon B, Suri A, Miller MJ, Unanue ER. Dendritic cells in islets of Langerhans constitutively present β cell-derived peptides bound to their class II MHC molecules. Proceedings of the National Academy of Sciences. 2008;105(16):6121-6.
