# Supplementary material for: Hybrid computational modeling demonstrates the utility of simulating complex cellular networks in type 1 diabetes
Source: PLoS Comput Biol. 2021 Sep 27;17(9):e1009413. doi: 10.1371/journal.pcbi.1009413 (PMC8496846; doi:10.1371/journal.pcbi.1009413)
Supplement: S3 Table — Rules were described using pseudocode in NetLogo, and state variables associated with agents were identified by square brackets). (DOCX) [file pcbi.1009413.s003.docx]

**S3 Table.** Agent rules during T1D progression (rules were described using pseudocode in NetLogo, and state variables associated with agents were identified by square brackets)

| Main function:  if number of apoptotic β cells <$\boldsymbol{(x\times}\text{number of β cells}\boldsymbol{)}$, where $\boldsymbol{x\sim}\text{unif}\boldsymbol{(0.1,0.3)}$  repeat procedures 1-28  update simulation step $\boldsymbol{n}$: $\boldsymbol{n}\mathbf{=}\boldsymbol{n}\mathbf{+1}$  note: 1 stimulation step represents 1 hour in T1D progression  else  stop simulation  end if |
| --- |
| procedure 1: pancreatic β cells are damaged and become apoptotic β cells  $\boldsymbol{x}$: number of damaged β cells  for $\boldsymbol{i=1}\text{to}\boldsymbol{x}$  set the state variable [damagedBetaCell] to 1 for $\boldsymbol{i}_{\boldsymbol{th}}$ β cells  assign a state variable [lifespanOfDamagedBetaCell] for $\boldsymbol{i}_{\boldsymbol{th}}$ β cells  end for  end procedure 1  procedure 2: apoptotic β cells release antigens  ${\boldsymbol{\beta}_{\boldsymbol{AP}}\boldsymbol{\vert}}_{\boldsymbol{t=n}}$: number of apoptotic β cells at step $\boldsymbol{n}$  $\boldsymbol{x\sim}\mathcal{N(}\boldsymbol{\mu,}\boldsymbol{\sigma}^{\boldsymbol{2}}\boldsymbol{)}$, where $\boldsymbol{\mu}$: mean value of released antigens; $\boldsymbol{\sigma:0.06\times\mu}$  for $\boldsymbol{i=1}\text{to} \boldsymbol{N}_{\boldsymbol{A\beta}}\boldsymbol{(n)}$  ask $\boldsymbol{i}_{\boldsymbol{th}}$ apoptotic β cells: create $\boldsymbol{x}$ antigen instances  end for  end procedure 2  procedure 3: apoptotic β cells die  $\boldsymbol{j}_{\boldsymbol{1}}$: the amount of time (simulation steps) that apoptotic β cells have been lived for  for $\boldsymbol{i=1}\text{to} \boldsymbol{N}_{\boldsymbol{A\beta}}\boldsymbol{(n)}$  update $\boldsymbol{j}_{\boldsymbol{1}}$: $\boldsymbol{j}_{\boldsymbol{1}}\mathbf{=}\boldsymbol{j}_{\boldsymbol{1}}\mathbf{+1}$ for the $\boldsymbol{i}_{\boldsymbol{th}}$ apoptotic β cell  if $\boldsymbol{j}_{\boldsymbol{1}}\boldsymbol{=}$ [lifespanOfDamagedBetaCell]  force $\boldsymbol{i}_{\boldsymbol{th}}$ apoptotic β cell to die  reset corresponding state variables of binding CTLs  end if  end for  end procedure 3  procedure 4: resident dendritic cells engulf antigens and become antigen-presenting cells (APCs)  $\boldsymbol{N}_{\boldsymbol{dc}}\boldsymbol{(n)}$: number of dendritic cells at step $\boldsymbol{n}$  $\boldsymbol{x}_{\boldsymbol{1}}$: maximum number of engulfed antigens by dendritic cells  $\boldsymbol{x}_{\boldsymbol{2}}$: 20% for the percentage of resident dendritic cells becoming APCs  for $\boldsymbol{i=1}\mathbf{to} \boldsymbol{N}_{\boldsymbol{dc}}\boldsymbol{(n)}$  if $\boldsymbol{i}_{\boldsymbol{th}}$ dendritic cell is a resident dendritic cell  check the eight surrounding patches  check the antigen type on the patches  if the number of antigens on $\boldsymbol{i}_{\boldsymbol{th}}$ dendritic cell <$\boldsymbol{x}_{\boldsymbol{1}}$  assign [dendriticCellBindingToAntigen] to 1  set [antigenBindingToDC] to 1 for the antigens  end if  end if  set $\boldsymbol{x}_{\boldsymbol{2}}$ of dendritic cells with [dendriticCellBindingToAntigen = 1] to APCs  end for  end procedure 4  procedure 5: circulating dendritic cells are recruited within the pancreas  $\boldsymbol{x}$: ${\mathbf{DC}_{\mathbf{r}}\mathbf{\vert}}_{\mathbf{t=n}}\mathbf{-}{\mathbf{DC}_{\mathbf{r}}\mathbf{\vert}}_{\mathbf{t=n-1}}\mathbf{=}\mathbf{k}_{\mathbf{r}}{\boldsymbol{\beta}_{\mathbf{AP}}\mathbf{\vert}}_{\mathbf{t=n}}\mathbf{DC}_{\mathbf{s}}$  where ${\mathbf{DC}_{\mathbf{r}}\mathbf{\vert}}_{\mathbf{t=n}}$: number of dendritic cells at simulation step $\boldsymbol{n}$  ${\mathbf{DC}_{\mathbf{r}}\mathbf{\vert}}_{\mathbf{t=n-1}}$: number of dendritic cells at simulation step $\boldsymbol{n-1}$  $\mathbf{k}_{\mathbf{r}}$: 0.65 for recruitment rate  $\boldsymbol{N}_{\boldsymbol{dcincir}}\boldsymbol{(n)}$: number of dendritic cells in circulation at simulation step $\boldsymbol{n}$  ${\boldsymbol{\beta}_{\boldsymbol{AP}}\boldsymbol{\vert}}_{\boldsymbol{t=n}}$: number of apoptotic β cells at simulation step $\boldsymbol{n}$  $\boldsymbol{j}_{\boldsymbol{2}}$: the amount of time that dendritic cells have stayed in the circulation region  create $\boldsymbol{x}$ dendritic cells in the circulation region  for $\boldsymbol{i=1}\mathbf{to} \boldsymbol{N}_{\boldsymbol{dcincir}}\boldsymbol{(n)}$  assign [dendriticCellInCirculation] to 1 for $\boldsymbol{i}_{\boldsymbol{th}}$ dendritic cell  update $\boldsymbol{j}_{\boldsymbol{2}}$: $\boldsymbol{j}_{\boldsymbol{2}}\mathbf{=}\boldsymbol{j}_{\boldsymbol{2}}\mathbf{+1}$ for $\boldsymbol{i}_{\boldsymbol{th}}$ dendritic cell  end for  end procedure 5  procedure 6: dendritic cells migrate from circulation to islets  $\mathbf{t}_{\boldsymbol{dccirist}}$: the time required for dendritic cells migrating from circulation to islets  for $\boldsymbol{i=1}\mathbf{to} \boldsymbol{N}_{\boldsymbol{dcincir}}\boldsymbol{(n)}$  if $\boldsymbol{j}_{\boldsymbol{2}}$: of $\boldsymbol{i}_{\boldsymbol{th}}$dendritic cell = $\mathbf{t}_{\boldsymbol{dccirist}}$  move to pancreatic islets  assign [dendriticCellInIselt] to 1 for $\boldsymbol{i}_{\boldsymbol{th}}$ dendritic cell  reset [dendriticCellInCirculation] to 0  reset $\boldsymbol{j}_{\boldsymbol{2}}$ to 0 for $\boldsymbol{i}_{\boldsymbol{th}}$ dendritic cell  end if  end for  end procedure 6  procedure 7: dendritic cells move in islets following Brownian motion  $\boldsymbol{N}_{\boldsymbol{dcinist}}\left( \boldsymbol{n} \right)\boldsymbol{:}$ number of dendritic cells in islets at simulation step $\boldsymbol{n}$  for $\boldsymbol{i=1}\mathbf{to} \boldsymbol{N}_{\boldsymbol{dcinist}}\boldsymbol{(n)}$  if location of $\boldsymbol{i}_{\boldsymbol{th}}$ dendritic cell is in pancreatic lymphatics  set [heading] of $\boldsymbol{i}_{\boldsymbol{th}}$ dendritic cell to  [heading] of $\boldsymbol{i}_{\boldsymbol{th}}$ dendritic cell + 180  else  choose [heading] of $\boldsymbol{i}_{\boldsymbol{th}}$ dendritic cell from $\mathbf{unif}\left( \boldsymbol{0, 360} \right)$  move $\boldsymbol{x}$steps, where $\boldsymbol{x}$: speed of dendritic cells in islets  end if  end for  end procedure 7  procedure 8: recruited dendritic cells engulf antigens and become APCs  $\boldsymbol{x}_{\boldsymbol{1}}$: maximum number of engulfed antigens by dendritic cells  for $\boldsymbol{i}\boldsymbol{=1}\mathbf{to} \boldsymbol{N}_{\boldsymbol{dc}}\boldsymbol{(n)}$  if $\boldsymbol{i}_{\boldsymbol{th}}$ dendritic cell is NOT a resident dendritic cell  check the eight surrounding patches  check the antigen type on the patches  if the number of antigens on $\boldsymbol{i}_{\boldsymbol{th}}$ dendritic cell < $\boldsymbol{x}_{\boldsymbol{1}}$  assign [dendriticCellBindingToAntigen] to 1  assign [antigenBindingToDC] to 1 for the antigens  end if  end if  end for  end procedure 8  procedure 9: dendritic cells migrate from pancreatic islets to PLNs  $\boldsymbol{j}_{\boldsymbol{3}}$: the amount of time that dendritic cells have stayed in the pancreatic islets  $\boldsymbol{j}_{\boldsymbol{4}}$: the amount of time that dendritic cells have stayed in PLNs  for $\boldsymbol{i=1}\mathbf{to} \boldsymbol{N}_{\boldsymbol{dcinist}}\boldsymbol{(n)}$  update $\boldsymbol{j}_{\boldsymbol{3}}$: $\boldsymbol{j}_{\boldsymbol{3}}\mathbf{=}\boldsymbol{j}_{\boldsymbol{3}}\mathbf{+1}$for $\boldsymbol{i}_{\boldsymbol{th}}$ dendritic cell in islets  if $\boldsymbol{j}_{\boldsymbol{3}}\boldsymbol{=}$ [timeRequiredForDCsMigratingFromIsletsToPLN]  move to PLN region  assign [dendriticCellInPLN] to 1 for $\boldsymbol{i}_{\boldsymbol{th}}$ dendritic cell  reset [dendriticCellInIslet] to 0 for $\boldsymbol{i}_{\boldsymbol{th}}$ dendritic cell  reset $\boldsymbol{j}_{\boldsymbol{3}}$ to 0 for $\boldsymbol{i}_{\boldsymbol{th}}$ dendritic cell  update $\boldsymbol{j}_{\boldsymbol{4}}$: $\boldsymbol{j}_{\boldsymbol{4}}\mathbf{=}\boldsymbol{j}_{\boldsymbol{4}}\mathbf{+1}$ for $\boldsymbol{i}_{\boldsymbol{th}}$ dendritic cell  end if  end for  end procedure 9  procedure 10: generation of naïve CD8^+^T cells in PLNs  $\boldsymbol{x}$: initial counts of naïve CD8^+^T cells  if number of naïve CD8^+^T cells < $\boldsymbol{x}$  create ($\boldsymbol{x}$ - number of naïve CD8^+^T cells) naïve CD8^+^T cells  assign [lifespanOfNaiveCD8Tcell] to naïve CD8^+^T cells  end for  end procedure 10  procedure 11: naïve CD8^+^T cells move within PLNs following Brownian motion  $\boldsymbol{N}_{\boldsymbol{ncd}\boldsymbol{8}\boldsymbol{inPLN}}\boldsymbol{(n)}$: number of naïve CD8^+^T cells in PLNs at simulation step $\boldsymbol{n}$  for $\boldsymbol{i}\boldsymbol{=1}\mathbf{to} \boldsymbol{N}_{\boldsymbol{ncd}\boldsymbol{8}\boldsymbol{inPLN}}\boldsymbol{(n)}$  if location of $\boldsymbol{i}_{\boldsymbol{th}}$ naïve CD8^+^T cell is in PLNs  set heading of $\boldsymbol{i}_{\boldsymbol{th}}$ naïve CD8^+^T cell to $\mathbf{unif}\left( \boldsymbol{0, 360} \right)$  move $\boldsymbol{x}$ steps, where $\boldsymbol{x}$: speed of naïve CD8^+^T cells in PLNs  else  move back to PLNs  end if  end for  end procedure 11  procedure 12: dendritic cells move within PLNs following Brownian motion  $\boldsymbol{N}_{\boldsymbol{dcinPLN}}\boldsymbol{(n)}$: number of dendritic cells in PLNs at simulation step $\boldsymbol{n}$  for $\boldsymbol{i}\boldsymbol{=1}\mathbf{to} \boldsymbol{N}_{\boldsymbol{dcinPLN}}\boldsymbol{(n)}$  if location of $\boldsymbol{i}_{\boldsymbol{th}}$ dendritic cell is in PLNs  set heading of $\boldsymbol{i}_{\boldsymbol{th}}$ dendritic cell to $\mathbf{unif}\left( \boldsymbol{0, 360} \right)$  move $\boldsymbol{x}$ steps, where $\boldsymbol{x}$: speed of dendritic cells in PLNs  else  move back to PLNs  end if  end for  end procedure 12  procedure 13: dendritic cells present antigens to naïve CD8^+^T cells  $\boldsymbol{x}_{\boldsymbol{1}}$: maximum number of naïve CD8^+^T cells that can bind to one dendritic cell  $\boldsymbol{x}_{\boldsymbol{2}}$: maximum number of dendritic cells that can bind to one naïve CD8^+^T cell  for $\boldsymbol{i}\boldsymbol{=1}\mathbf{to} \boldsymbol{N}_{\boldsymbol{dcinPLN}}\boldsymbol{(n)}$  check eight surrounding patches  check antigen type on the patches  if number of naïve CD8^+^T cell on $\boldsymbol{i}_{\boldsymbol{th}}$ APC < $\boldsymbol{x}_{\boldsymbol{1}}$  if the number of dendritic cells on naïve CD8^+^T cell< $\boldsymbol{x}_{\boldsymbol{2}}$  assign [DCsBindingToNaiveCD8Tcell] to 1 for $\boldsymbol{i}_{\boldsymbol{th}}$ APC  assign [naiveCD8TcellBindingToDCs] to 1  end if  end if  end for  end procedure 13  procedure 14: naïve CD8^+^T cells are activated  $\boldsymbol{j}_{\boldsymbol{5}}$: the amount of time that naïve CD8^+^T cells have been bound to dendritic cells  $\boldsymbol{x\sim}\mathbf{unif}\boldsymbol{(2, 24)}$  for $\boldsymbol{i}\boldsymbol{=1}\mathbf{to} \boldsymbol{N}_{\boldsymbol{ncd}\boldsymbol{8inPLN}}\boldsymbol{(n)}$  if $\boldsymbol{i}_{\boldsymbol{th}}$ naïve CD8^+^T cell with [naiveCD8TcellBindingToDCs = 1]  update $\boldsymbol{j}_{\boldsymbol{5}}$: $\boldsymbol{j}_{\boldsymbol{5}}\mathbf{=}\boldsymbol{j}_{\boldsymbol{5}}\mathbf{+1}$ for $\boldsymbol{i}_{\boldsymbol{th}}$ naïve CD8^+^T cell in PLNs  if $\boldsymbol{j}_{\boldsymbol{5}}\boldsymbol{=x}$  assign [naiveCD8TcellEnterDifferentiationCycle] to 1  reset $\boldsymbol{j}_{\boldsymbol{5}}$ to 0 for $\boldsymbol{i}_{\boldsymbol{th}}$ naïve CD8^+^T cell  end if  end if  end for  end procedure 14  procedure 15: differentiation of naïve CD8^+^T cells in PLNs  $\boldsymbol{j}_{\boldsymbol{6}}$: the amount of time that naïve CD8^+^T cells have stayed in the differentiation cycle  $\boldsymbol{x}\boldsymbol{\sim}\mathbf{unif}\boldsymbol{(4,8)}$  for $\boldsymbol{i}\boldsymbol{=1}\mathbf{to} \boldsymbol{N}_{\boldsymbol{ncd}\boldsymbol{8}\boldsymbol{inPLN}}\boldsymbol{(n)}$  update $\boldsymbol{j}_{\boldsymbol{6}}$: $\boldsymbol{j}_{\boldsymbol{6}}\mathbf{=}\boldsymbol{j}_{\boldsymbol{6}}\mathbf{+1}$ for $\boldsymbol{i}_{\boldsymbol{th}}$ naïve CD8^+^T cell in PLNs  if $\boldsymbol{j}_{\boldsymbol{6}}\boldsymbol{=x}$  create 1 activated CD8^+^T cell in PLNs  assign [lifespanOfActivatedCD8TcellInPLN] to activated CD8^+^T cell  assign [activatedCD8TcellInPLN] to 1 for activated CD8^+^T cell  end if  end for  end procedure 15  procedure 16: activated CD8^+^T cells migrate from PLNs to islets  $\boldsymbol{x}$: 120h for 5 days (120 hours) that CD8^+^T cells migrate  $\boldsymbol{j}_{\boldsymbol{7}}$: the amount of time that activated CD8^+^T cells have stayed in PLNs  for $\boldsymbol{i}\boldsymbol{=1}\mathbf{to} \boldsymbol{N}_{\boldsymbol{acd}\boldsymbol{8}\boldsymbol{inPLN}}\boldsymbol{(n)}$  update $\boldsymbol{j}_{\boldsymbol{7}}$: $\boldsymbol{j}_{\boldsymbol{7}}\mathbf{=}\boldsymbol{j}_{\boldsymbol{7}}\mathbf{+1}$ for $\boldsymbol{i}_{\boldsymbol{th}}$ activated CD8^+^T cell in PLNs  if $\boldsymbol{j}_{\boldsymbol{7}}\boldsymbol{=x}$  move to pancreatic islets  reset [activatedCD8TcellInPLN] to 0 for $\boldsymbol{i}_{\boldsymbol{th}}$ activated CD8^+^T cell  assign [activatedCD8TcellInIslet] to 1 for $\boldsymbol{i}_{\boldsymbol{th}}$ activated CD8^+^T cell  reset $\boldsymbol{j}_{\boldsymbol{7}}$ to 0 for $\boldsymbol{i}_{\boldsymbol{th}}$ activated CD8^+^T cell  end for  end procedure 16  procedure 17: activated CD8^+^T cells move in islets  $\boldsymbol{N}_{\boldsymbol{acd}\boldsymbol{8}\boldsymbol{inist}}\boldsymbol{(n)}$: number of activated CD8^+^T cells in islets at simulation step $\boldsymbol{n}$  for $\boldsymbol{i}\boldsymbol{=1}\mathbf{to} \boldsymbol{N}_{\boldsymbol{acd}\boldsymbol{8}\boldsymbol{inist}}\boldsymbol{(n)}$  if location of $\boldsymbol{i}_{\boldsymbol{th}}$ activated CD8^+^T cell is in PLNs  set [heading] of $\boldsymbol{i}_{\boldsymbol{th}}$ activated CD8^+^T cell =  [heading] of $\boldsymbol{i}_{\boldsymbol{th}}$ activated CD8^+^T cell + 180 else  set [heading] of $\boldsymbol{i}_{\boldsymbol{th}}$ activated CD8^+^T cell to $\mathbf{unif}\left( \boldsymbol{0, 360} \right)$  move $\boldsymbol{x}$ steps, where $\boldsymbol{x}$: speed of activated CD8^+^T cells in islets  end if  end for  end procedure 17  procedure 18: activated CD8^+^T cells improve antigen presentation of dendritic cells  for $\boldsymbol{i}\boldsymbol{=1}\mathbf{to} \boldsymbol{N}_{\boldsymbol{acd}\boldsymbol{8}\boldsymbol{inist}}\boldsymbol{(n)}$  check eight surrounding patches  check type of agents on patches  if any dendritic cell on patches  if dendritic cell engulfed antigens  assign [dendriticCellPresentAntigen] to 1 for dendritic cell  end if  end if  end for  end procedure 18  procedure 19: activated CD8^+^ T cells become CTLs in islets  $\boldsymbol{x\sim}\mathcal{N(}\boldsymbol{0.8,0.048)}$  for $\boldsymbol{i}\boldsymbol{=1}\mathbf{to} \left( \boldsymbol{x\times N}_{\boldsymbol{acd}\boldsymbol{8}\boldsymbol{inist}}\boldsymbol{(n)} \right)$  change agent type to CTL  assign [lifespanOfCTLInIslet] to CTL  end for  end procedure 19  procedure 20: CTLs move in islets following Brownian motion  $\boldsymbol{N}_{\boldsymbol{CTLinist}}\boldsymbol{(n)}$: number of CTLs in islets at simulation step $\boldsymbol{n}$  for $\boldsymbol{i}\boldsymbol{=1}\mathbf{to} \boldsymbol{N}_{\boldsymbol{CTLinist}}\boldsymbol{(n)}$  if location of $\boldsymbol{i}_{\boldsymbol{th}}$ CTL is in PLNs  set [heading] of $\boldsymbol{i}_{\boldsymbol{th}}$ CTL = [heading] of $\boldsymbol{i}_{\boldsymbol{th}}$ CTL + 180 else  set [heading] of $\boldsymbol{i}_{\boldsymbol{th}}$ CTL to $\mathbf{unif}\left( \boldsymbol{0, 360} \right)$  move $\boldsymbol{x}$ steps, where $\boldsymbol{x}$: speed of CTLs in islets  end if  end for  end procedure 20  procedure 21: CTLs bind to β cells  $\boldsymbol{x}_{\boldsymbol{1}}$: maximum number of β cells that can bind to CTL  $\boldsymbol{x}_{\boldsymbol{2}}$: maximum number of CTLs that can bind to β cell  for $\boldsymbol{i}\boldsymbol{=1}\mathbf{to} \boldsymbol{N}_{\boldsymbol{CTLinist}}\boldsymbol{(n)}$  check eight surrounding patches  check type of agents on patches  if any pancreatic β cell on patches  if number of β cells binding to $\boldsymbol{i}_{\boldsymbol{th}}$ CTL < $\boldsymbol{x}_{\boldsymbol{1}}$ if number of CTLs binding to β cell < $\boldsymbol{x}_{\boldsymbol{2}}$  assign [CTLBindToBetaCell] to 1 for i_th_ CTL  assign [BetaCellBindToCTL] to 1 for the β cell  end if  end if  end if  end for  end procedure 21  procedure 22: pancreatic β cells become infected after binding to CTLs  $\boldsymbol{N}_{\boldsymbol{\beta}}\boldsymbol{(n)}$: number of β cells in islets at simulation step $\boldsymbol{n}$  for $\boldsymbol{i}\boldsymbol{=1}\mathbf{to} \boldsymbol{N}_{\boldsymbol{\beta}}\boldsymbol{(n)}$  if $\boldsymbol{i}_{\boldsymbol{th}}$ β cell binds to CTL  assign [damagedBetaCell] to 1 for $\boldsymbol{i}_{\boldsymbol{th}}$ β cell  assign [lifespanOfDamagedBetaCell] to 1 for $\boldsymbol{i}_{\boldsymbol{th}}$ β cell  end if  end for  end procedure 22  procedure 23: CTLs proliferate in islets  $\boldsymbol{N}_{\boldsymbol{CTLbindto\beta}}\boldsymbol{(n)}$: number of CTLs that bind to β cells at simulation step $\boldsymbol{n}$  $\boldsymbol{x}_{\boldsymbol{1}}\boldsymbol{\sim}\mathbf{unif}\boldsymbol{(0.154,0.238)}$  $\boldsymbol{x}_{\boldsymbol{2}}$: 120h required to be able to proliferate  $\boldsymbol{x}_{\boldsymbol{3}}\boldsymbol{\sim}\mathbf{unif}\boldsymbol{(4,7)}$  $\boldsymbol{j}_{\boldsymbol{8}}$: the time that CTL has stayed in the differentiation cycle  for $\boldsymbol{i}\boldsymbol{=1}\mathbf{to} \boldsymbol{(x}_{\boldsymbol{1}}\boldsymbol{\times}\boldsymbol{N}_{\boldsymbol{CTLbindto\beta}}\boldsymbol{(n))}$  assign [CTLStartToDifferentiation] to 1 for $\boldsymbol{i}_{\boldsymbol{th}}$ β cell  update $\boldsymbol{j}_{\boldsymbol{8}}$: $\boldsymbol{j}_{\boldsymbol{8}}\mathbf{=}\boldsymbol{j}_{\boldsymbol{8}}\mathbf{+1}$ for $\boldsymbol{i}_{\boldsymbol{th}}$ β cell  if $\boldsymbol{j}_{\boldsymbol{8}}\boldsymbol{=}\boldsymbol{x}_{\boldsymbol{2}}$  create $\boldsymbol{x}_{\boldsymbol{3}}$ CTLs  end if  end for  end procedure 23  procedure 24: dendritic cells die in PLNs  $\boldsymbol{j}_{\boldsymbol{9}}$: the amount of time that dendritic cells have stayed in PLNs  $\boldsymbol{x\sim}\mathbf{unif}\boldsymbol{(48,72)}$  for $\boldsymbol{i}\boldsymbol{=1}\mathbf{to} \boldsymbol{N}_{\boldsymbol{dcinPLN}}\boldsymbol{(n)}$  update $\boldsymbol{j}_{\boldsymbol{9}}$: $\boldsymbol{j}_{\boldsymbol{9}}\mathbf{=}\boldsymbol{j}_{\boldsymbol{9}}\mathbf{+1}$ for $\boldsymbol{i}_{\boldsymbol{th}}$ dendritic cell in PLNs  if $\boldsymbol{j}_{\boldsymbol{9}}\boldsymbol{=x}$  force $\boldsymbol{i}_{\boldsymbol{th}}$ dendritic cell to disappear  end if  end for  end procedure 24  procedure 25: CTLs die in islets  $\boldsymbol{j}_{\boldsymbol{10}}$: the amount of time that CTLs have stayed in islets  $\boldsymbol{x}$: the lifespan of CTLs in islets  for $\boldsymbol{i}\boldsymbol{=1}\mathbf{to} \boldsymbol{N}_{\boldsymbol{CTLinist}}\boldsymbol{(n)}$  update $\boldsymbol{j}_{\boldsymbol{10}}$: $\boldsymbol{j}_{\boldsymbol{10}}\mathbf{=}\boldsymbol{j}_{\boldsymbol{10}}\mathbf{+1}$ for $\boldsymbol{i}_{\boldsymbol{th}}$ CTL  if $\boldsymbol{j}_{\boldsymbol{10}}\boldsymbol{=x}$  force $\boldsymbol{i}_{\boldsymbol{th}}$ CTL to disappear  end if  end for  end procedure 25  procedure 26: activated CD8^+^ T cells die in islets  $\boldsymbol{N}_{\boldsymbol{acd}\boldsymbol{8}\boldsymbol{inist}}\boldsymbol{(n)}$: number of activated CD8^+^ T cells in islets at simulation step $\boldsymbol{n}$  $\boldsymbol{j}_{\boldsymbol{11}}$: the amount of time that activated CD8^+^ T cells have stayed in islets  $\boldsymbol{x}$: the lifespan of activated CD8^+^ T cells in islets  for $\boldsymbol{i}\boldsymbol{=1}\mathbf{to} \boldsymbol{N}_{\boldsymbol{acd}\boldsymbol{8}\boldsymbol{inist}}$(n)  update $\boldsymbol{j}_{\boldsymbol{11}}$: $\boldsymbol{j}_{\boldsymbol{11}}\mathbf{=}\boldsymbol{j}_{\boldsymbol{11}}\mathbf{+1}$ for $\boldsymbol{i}_{\boldsymbol{th}}$ CTL  if $\boldsymbol{j}_{\boldsymbol{11}}$ = $\boldsymbol{x}$  force $\boldsymbol{i}_{\boldsymbol{th}}$ activated CD8^+^ T cell to disappear  end if  end for  end procedure 26  procedure 27: naïve CD8^+^ T cells egress from PLNs  $\boldsymbol{N}_{\boldsymbol{ncd}\boldsymbol{8}\boldsymbol{inPLN}}\boldsymbol{(n)}$: number of naïve CD8^+^ T cells in PLNs  $\boldsymbol{j}_{\boldsymbol{12}}$: the amount of time that $\boldsymbol{i}_{\boldsymbol{th}}$ naïve CD8^+^ T cell has stayed in PLNs  $\boldsymbol{x}$: lifespan of naïve CD8^+^ T cell in PLNs  for $\boldsymbol{i}\boldsymbol{=1}\mathbf{to} \boldsymbol{N}_{\boldsymbol{acd}\boldsymbol{8}\boldsymbol{inist}}\boldsymbol{(n)}$  update $\boldsymbol{j}_{\boldsymbol{12}}$: $\boldsymbol{j}_{\boldsymbol{12}}\mathbf{=}\boldsymbol{j}_{\boldsymbol{12}}\mathbf{+1}$ for $\boldsymbol{i}_{\boldsymbol{th}}$ naïve CD8^+^ T cell in PLNs  if $\boldsymbol{j}_{\boldsymbol{12}}$ = $\boldsymbol{x}$  force $\boldsymbol{i}_{\boldsymbol{th}}$ naïve CD8^+^ T cell in PLNs to disappear  end if  end for  end procedure 27  procedure 28: pancreatic β cells proliferate in islets  $\boldsymbol{x}_{\boldsymbol{1}}$: length of $\boldsymbol{i}_{\boldsymbol{th}}$ β cell staying in a quiescence period at simulation step 0  $\boldsymbol{x}_{\boldsymbol{2}}$:${\boldsymbol{T}_{\boldsymbol{\beta}_{\boldsymbol{i}}}\boldsymbol{\vert}}_{\boldsymbol{t=n}}\boldsymbol{=}\left( \boldsymbol{1-}\frac{{\boldsymbol{\beta}_{\boldsymbol{AP}}\boldsymbol{\vert}}_{\boldsymbol{t=n}}}{\boldsymbol{\beta}_{\boldsymbol{init}}} \right)^{\boldsymbol{\gamma}}\boldsymbol{\cdot}{\boldsymbol{T}_{\boldsymbol{\beta}_{\boldsymbol{i}}}\boldsymbol{\vert}}_{\boldsymbol{t=0}}$  ${\boldsymbol{T}_{\boldsymbol{\beta}_{\boldsymbol{i}}}\boldsymbol{\vert}}_{\boldsymbol{t=n}}$: required quiescence period for the $\boldsymbol{i}_{\boldsymbol{th}}$ β cell at step $\boldsymbol{n}$  ${\boldsymbol{T}_{\boldsymbol{\beta}_{\boldsymbol{i}}}\boldsymbol{\vert}}_{\boldsymbol{t=0}}$: required quiescence period for the $\boldsymbol{i}_{\boldsymbol{th}}$ β cell at step 0  $\boldsymbol{\beta}_{\boldsymbol{init}}$: number of β cells at simulation step 0  $\boldsymbol{\gamma}$: 1 shows estimated degree of glucose metabolism  $\boldsymbol{x}_{\boldsymbol{3}}$*:* 24h for quiescence period when 70% of apoptotic β cells appear in the simulation  $\boldsymbol{j}_{\boldsymbol{13}}$: length of $\boldsymbol{i}_{\boldsymbol{th}}$ β cell staying in quiescence period when step n > 0  $\boldsymbol{j}_{\boldsymbol{14}}$: length of $\boldsymbol{i}_{\boldsymbol{th}}$ β cell staying in differentiation cycle  for $\boldsymbol{i}\boldsymbol{=1}\mathbf{to} \boldsymbol{N}_{\boldsymbol{\beta inist}}\boldsymbol{(n)}$  if pancreatic β cells is not damaged  update $\boldsymbol{j}_{\boldsymbol{13}}$: $\boldsymbol{j}_{\boldsymbol{13}}\mathbf{=}\boldsymbol{j}_{\boldsymbol{13}}\mathbf{+1}$ for $\boldsymbol{i}_{\boldsymbol{th}}$ β cell in PLNs  if ($\boldsymbol{j}_{\boldsymbol{13}}$ + $\boldsymbol{x}_{\boldsymbol{1}}$) = $\boldsymbol{x}_{\boldsymbol{2}}$  assign [betaCellUndergoQuiescencePeriod] to 0 for $\boldsymbol{i}_{\boldsymbol{th}}$β cell  reset $\boldsymbol{j}_{\boldsymbol{13}}$ to 0 for $\boldsymbol{i}_{\boldsymbol{th}}$β cell  update $\boldsymbol{j}_{\boldsymbol{14}}$: $\boldsymbol{j}_{\boldsymbol{14}}\mathbf{=}\boldsymbol{j}_{\boldsymbol{14}}\mathbf{+1}$  if $\boldsymbol{j}_{\boldsymbol{14}}$ = $\boldsymbol{x}_{\boldsymbol{3}}$  create 1 new β cell  end if  end if  end if  end for  end procedure 28 |
