# Supplementary material for: Hybrid computational modeling demonstrates the utility of simulating complex cellular networks in type 1 diabetes
Source: PLoS Comput Biol. 2021 Sep 27;17(9):e1009413. doi: 10.1371/journal.pcbi.1009413 (PMC8496846; doi:10.1371/journal.pcbi.1009413)
Supplement: S4 Table — Parameter ranges used for local sensitivity analysis and Latin hypercube sampling (LHS) are tabulated below. By consulting with the field experts and cited papers, the ranges were found in the literature (in this case literature does are not necessarily mean about NOD mouse model since these parameters are defined as unknown) which were then employed for unknown parameters (i.e. Ranges used for LHS) for the purpose of initial simulation of the model. The ranges determined by the field experts are marked using asterisks. (DOCX) [file pcbi.1009413.s004.docx]

**S4 Table.** Unknown parameters in the ABM simulations. Parameter ranges used for local sensitivity analysis (LSA) and Latin hypercube sampling (LHS) are tabulated below. By consulting with the field experts and cited papers, the ranges were found in the literature (in this case literature does are not necessarily mean about NOD mouse model since these parameters are defined as unknown) which were then employed for unknown parameters (i.e. Ranges used for LHS) for the purpose of initial simulation of the model. The ranges determined by the field experts are marked using asterisks.

| Parameters | Default value | Unit | Ranges used for LSA | Ranges used for LHS |
| --- | --- | --- | --- | --- |
| Maximum number of β cells binding to CTLs | 3 | count | [2.4-3.6] | [1-10] [1,2] |
| Maximum number of CTLs binding to β cells | 4 | count | [3.2-4.8] | [1-10] [1,2] |
| Speed of DCs in islets | 4 | µm/hr | [3.2-4.8] | [1-60] [3] |
| Percentage of activated CD8^+^ T cells becoming CTLs | 0.8 | -- | [0.64-0.96] | [0-1] |
| Initial number of naïve CD8^+^ T cells | 15000 | count | [12-18] $\times$10^3^ | [10-100] $\times$10^3^ [4] |
| Average lifespan of apoptotic β cells | 6 | hr | [4.8-7.2] | [1-74] [5] |
| Maximum number of DCs binding to naïve CD8^+^ T cells | 5 | count | [4-6] | [1-10] [1,2] |
| Maximum number of naïve CD8^+^ T cells binding to DCs | 2 | count | [1.6-2.4] | [1-10] [1,2] |
| Speed of CTLs in islets | 2 | µm/hr | [1.6-2.4] | [1-60] [6] |
| Recruitment rate of DCs within the pancreas | 0.0065 | -- | [5.2-7.8]$\times$10^-3^ | [1-10]$\times$10^-3^ [7] |
| Speed of activated CD8^+^ T cells in islets | 5 | µm/hr | [4-6] | [1-60] [6] |
| Average lifespan of CTLs in islets | 120 | hr | [96-144] | [72-192] [8,9] |
| Initial number of damaged cells | 5 | count | [4-6] | [1-36] [10] |
| Speed of DCs in PLNs | 4 | µm/hr | [3.2-4.8] | [1-60] [3] |
| Number of released antigens by each apoptotic β cell | 3 | count | [2.4-3.6] | [1-12] [11] |
| Speed of naïve CD8^+^ T cells in PLNs | 4 | µm/hr | [3.2-4.8] | [1-60] [3] |
| Maximum number of antigens binding to DCs | 4 | count | [3.2-4.8] | [1-10] [1,2] |
| Average lifespan of activated CD8^+^ T cells in islets | 100 | hr | [80-120] | [72-192] [8,9] |
| Time required for activated CD8^+^ T cells movement in islets | 25 | hr | [20-30] | [10-100] [6] |
| Time required for CTLs movement in islets | 45 | hr | [36-54] | [10-100] [6] |
| Time required for DCs movement in islets | 320 | hr | [256-384] | [80-800] [3] |

**References**

1. Scherer A, Salathé M, Bonhoeffer S. High epitope expression levels increase competition between T cells. PLoS computational biology. 2006;2(8):e109.

2. Jobling MG, Yang Z, Kam WR, Lencer WI, Holmes RK. A single native ganglioside GM1-binding site is sufficient for cholera toxin to bind to cells and complete the intoxication pathway. MBio. 2012;3(6):e00401-12.

3. Calderon B, Suri A, Miller MJ, Unanue ER. Dendritic cells in islets of Langerhans constitutively present β cell-derived peptides bound to their class II MHC molecules. Proceedings of the National Academy of Sciences. 2008;105(16):6121-6.

4. den Braber I, Mugwagwa T, Vrisekoop N, Westera L, Mögling R, de Boer AB, et al. Maintenance of peripheral naive T cells is sustained by thymus output in mice but not humans. Immunity. 2012;36(2):288-97.

5. Breart B, Lemaître F, Celli S, Bousso P. Two-photon imaging of intratumoral CD8+ T cell cytotoxic activity during adoptive T cell therapy in mice. The Journal of clinical investigation. 2008;118(4):1390-7.

6. Coppieters K, Amirian N, von Herrath M. Intravital imaging of CTLs killing islet cells in diabetic mice. The Journal of clinical investigation. 2012;122(1):119-31.

7. Clare-Salzler M, Mullen Y. Marked dendritic cell-T cell cluster formation in the pancreatic lymph node of the non-obese diabetic mouse. Immunology. 1992;76(3):478.

8. Schmitz I, Krueger A, Baumann S, Schulze-Bergkamen H, Krammer PH, Kirchhoff S. An IL-2-dependent switch between CD95 signaling pathways sensitizes primary human T cells toward CD95-mediated activation-induced cell death. The Journal of Immunology. 2003;171(6):2930-6.

9. Joshi NS, Cui W, Chandele A, Lee HK, Urso DR, Hagman J, et al. Inflammation directs memory precursor and short-lived effector CD8+ T cell fates via the graded expression of T-bet transcription factor. Immunity. 2007;27(2):281-95.

10. Kurrer MO, Pakala SV, Hanson HL, Katz JD. β cell apoptosis in T cell-mediated autoimmune diabetes. Proceedings of the National Academy of Sciences. 1997;94(1):213-8.

11. Babad J, Geliebter A, DiLorenzo TP. T‐cell autoantigens in the non‐obese diabetic mouse model of autoimmune diabetes. Immunology. 2010;131(4):459-65.
