# Supplementary material for: Hybrid computational modeling demonstrates the utility of simulating complex cellular networks in type 1 diabetes
Source: PLoS Comput Biol. 2021 Sep 27;17(9):e1009413. doi: 10.1371/journal.pcbi.1009413 (PMC8496846; doi:10.1371/journal.pcbi.1009413)
Supplement: S5 Table — (DOCX) [file pcbi.1009413.s005.docx]

**S5 Table.** Computational costs for sensitivity analysis and therapy-directed simulations

|  | Total Task | Simulation runs for total task | Single-core time consumption* | Parallel computing* |
| --- | --- | --- | --- | --- |
| Local sensitivity analysis | $21$ | $21\times20\times9$ | 6 months | 4.5 days |
| Global sensitivity analysis | $5$ | $10\times1000$ | 7.2 months | 5.4 days |
| Therapy-directed simulations | $24$ | $24\times3380$ | 28.2 months | 21.2 days |

**Note:** For local sensitivity analysis, a total of 21 parameters was tested. 9 values including the default value were selected for each parameter, four on the left-hand side and four on the right-hand side of the default value. In each test, 20 simulation runs were implemented to capture the stochastic nature of ABM simulation. Global sensitivity analysis was implemented based on eFAST, and combinations of 5 parameters were incorporated into the ABM simulations. The total combinations of sampling points for eFAST were set to 1000, the samplings are shown in Fig. S3. Each combination was repeated by 10 simulation runs. A total number of simulation runs was divided into five tasks that were implemented simultaneously using parallel computing. For therapy-directed simulations, each test has approximately 169 tested points, and each tested point was implemented in 20 simulation runs to calculate the probability of T1D occurrence. 3 heatmaps for three parameters were generated for one-way therapy-directed analysis and 21 heatmaps (7×3, 7 times points for 3 parameter combinations) for two-way therapy-directed analysis, a total of 24 tests. For each combination, we tested the effectiveness of the proposed therapies from week 4 to week 16, with an interval of 2 weeks. In the above tests, each simulation run took approximately 1 hour on our local server (server-specific configuration: 4x Intel Xeon CPU E5-4650 2.1 GHz 48 cores with 192 GB of RAM)

*Time consumption is the time required for each analysis if an individual simulation run was implemented consecutively. However, we reduced time consumption significantly by means of parallel computing. For parallel computing, 40 simulation runs were implemented simultaneously on our local server.
