# Supplementary figures and images for: Hybrid computational modeling demonstrates the utility of simulating complex cellular networks in type 1 diabetes
Source: PLoS Comput Biol. 2021 Sep 27;17(9):e1009413. doi: 10.1371/journal.pcbi.1009413 (PMC8496846; doi:10.1371/journal.pcbi.1009413)

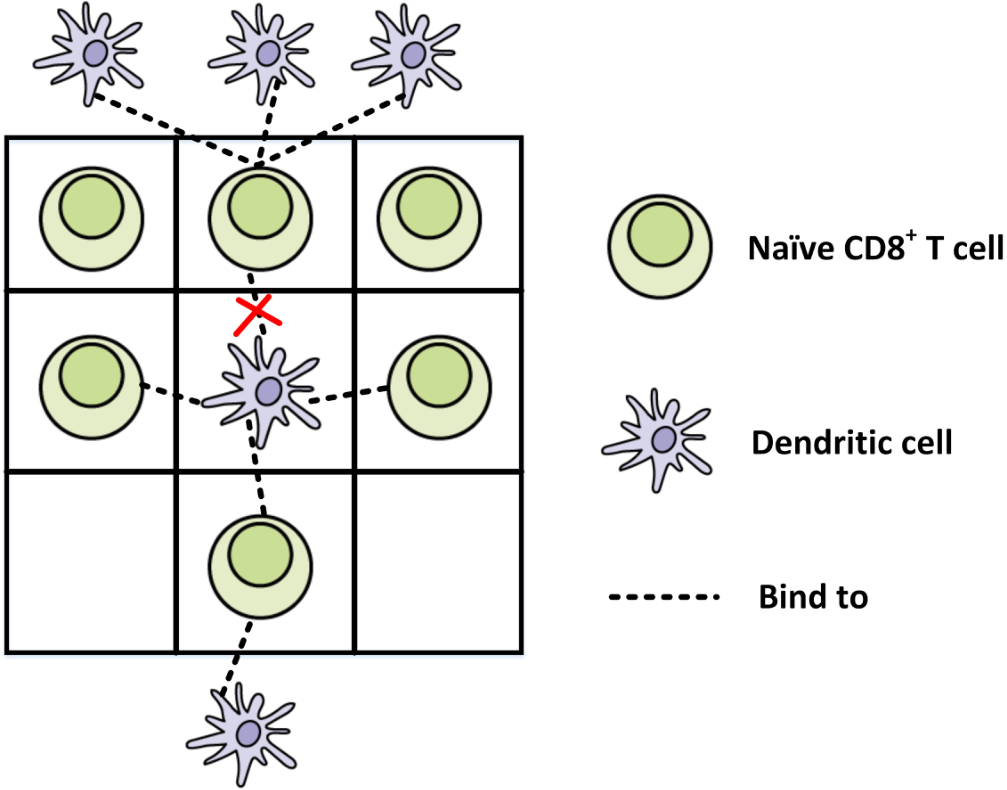

Supplement: S1 Fig — During the binding process, each APC (i.e. dendritic cell that has engulfed antigens) checked the surrounding eight patches. If naïve CD8+T cells appear on these eight patches, an APC checks the number of dendritic cells binding to each naïve CD8+T cell. A dendritic cell can bind to a naïve CD8+T cell if this naïve CD8+T cell has available binding sites for the dendritic cell (e.g. the naïve CD8+ T cell at the bottom of nine grids in S1 Fig). A dendritic cell can bind to multiple naïve CD8+ T cells until there are no available binding sites on the naïve CD8+ T cell (e.g. the naïve CD8+ T cell in the top of nine grids in S1 Fig). The number of binding sites on dendritic cells for naïve CD8+ T cells are determined by a state variable named the maximum number of naïve CD8+ T cells binding to DCs. (TIFF) [file pcbi.1009413.s006.tiff]

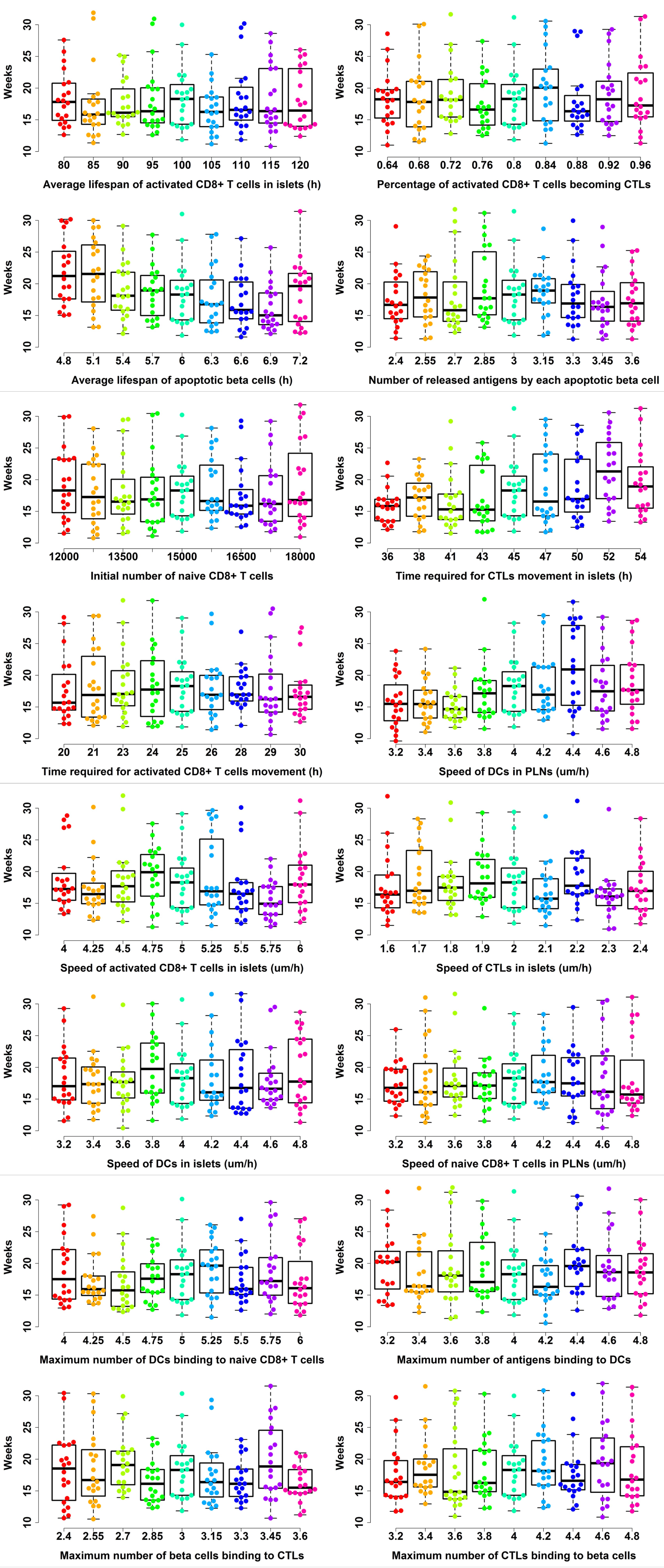

Supplement: S4 Fig — Discernible fluctuation in time required for developing overt T1D based upon selected values of parameters using local sensitivity analysis. The horizontal axis represents selected values of five input parameters in the spectrum of [Pd±30%×Pd], and the vertical axis denotes the time required for developing T1D. Box plots and raw data illustrate heterogeneities occurring during T1D for selected values. Black dotted lines outline lower and upper whiskers and black solid boxes show Q1, Q2, Q3 for first quartile, interquartile, and third quantile values for selected points within the spectrum of [Pd±30%×Pd]. For non-sensitive parameters, the p-values of F tests were greater than the predetermined significance level (e.g. α = 0.05). (TIFF) [file pcbi.1009413.s009.tiff]

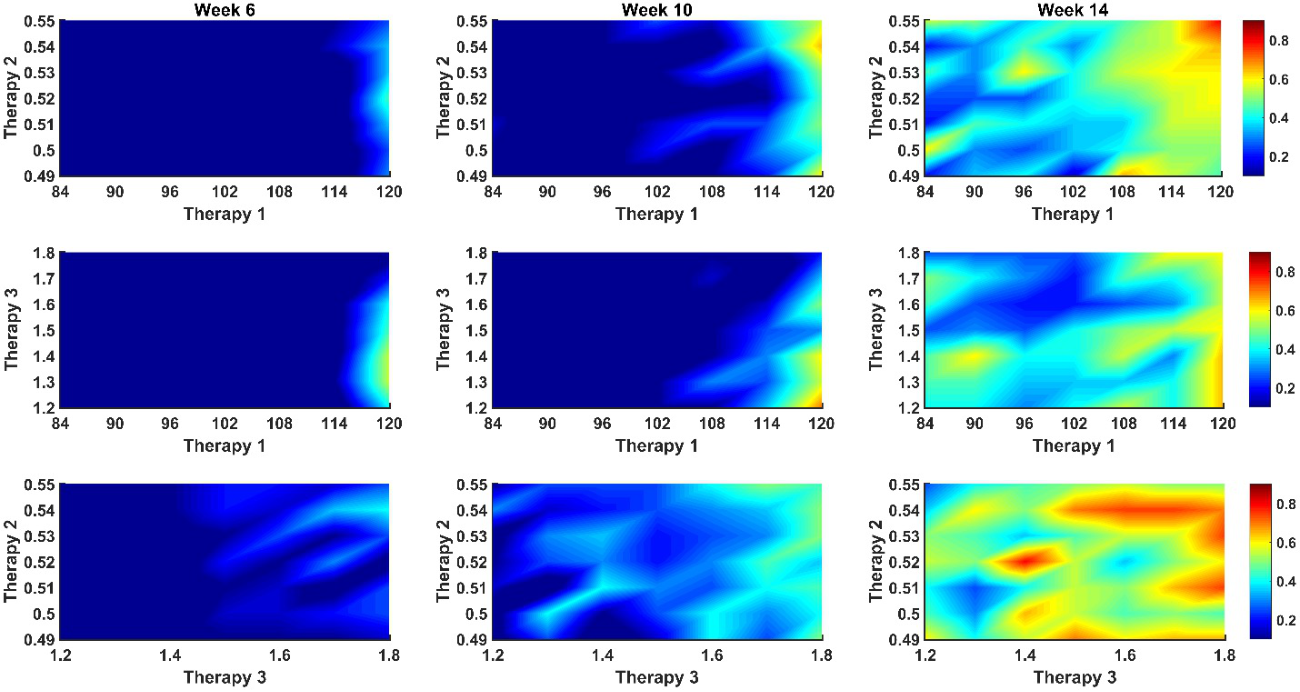

Supplement: S5 Fig — Therapy interventions were implemented based upon single dosage regimens starting at week 6 to week 14. Regions with red color represent a higher incidence of diabetes, and regions with blue color represent a lower incidence of diabetes. Therapy 1 represents a certain strategy that reduces the residence of CTLs in islets. Therapy 2 is described as an intervention that can inhibit DC infiltration into islets. Therapy 3 denotes a strategy that prohibits binding sites on DCs for naïve CD8+ T cells. (TIFF) [file pcbi.1009413.s010.tiff]
