# Supplementary material for: Hybrid computational modeling demonstrates the utility of simulating complex cellular networks in type 1 diabetes
Source: PLoS Comput Biol. 2021 Sep 27;17(9):e1009413. doi: 10.1371/journal.pcbi.1009413 (PMC8496846; doi:10.1371/journal.pcbi.1009413)
Supplement: Attachment — Submitted filename: Response to reviewers.docx [file pcbi.1009413.s012.docx]

**July 22, 2021**

**Response letter of [PCOMPBIOL-D-21-00374] – major revision**

Dear Reviewers and Editor:

We appreciate the constructive comments and suggestions from editors and reviewers that greatly improve the quality of the manuscript. We have done our best to address them and incorporate most of the suggestions made by the reviewers. In this revision, we have also made several major changes to further clarify and improve the contents of the submitted manuscript in light of both reviewers’ and editors’ comments:

1. Clarified the usage of statistical learning, machine learning, and some other confusing words used in the original manuscript
2. Presented the Materials and Methods section earlier than the Result section to illustrate the development of ABM
3. Moved other Materials and Methods parts from Supplementary Materials to the main text to illustrate the development of ABM, the analysis of model outputs, and therapy-directed simulation.
4. Added a high-level model abstract to the Materials and Methods section to illustrate the key performance metrics of the model
5. Added a diagram to illustrate how the agents developed are interacting and the level of “intelligence” of the agents

To respond to the comments raised by the reviewers, our point-by-point response follows. The changes that we made in the main text were highlighted in this response letter. Our response is in blue. The references we used below are also hyperlinked to the sources.

Sincerely yours,

Majid Jaberi-Douraki, PhD
Associate Professor
1DATA Consortium, Director

KSU FARAD Program, Director

Johnson Cancer Center, Affiliate

Computational Comparative Medicine

Applied and Biomedical Sciences

Department of Mathematics

Kansas State University Olathe

22201 W. Innovation Dr. Olathe, KS 66061-1304

**Response letter of [PCOMPBIOL-D-21-00374] – major revision**

**Editor Comments:**

As with all papers reviewed by the journal, your manuscript was reviewed by members of the editorial board and by several independent reviewers. In light of the reviews (below this email), we would like to invite the resubmission of a significantly-revised version that takes into account the reviewers' comments. In particular, both reviewers requested clarification about the role of AI/machine learning approaches in the development of the model and connecting the simulation results to experimental data. An example of confusing language is provided in the title itself, which mentions connecting "data science" and "statistical learning" -- my understanding is that statistical learning is part of data science. Also the phrase "deciphers the expediency" doesn't have a very clear meaning. I think the authors are trying to say "demonstrates the utility of."

**Response:**  Thank you for giving us the opportunity to revise the manuscript. In light of both editors’ and reviewers’ comments, we realized some keywords such as statistical/machine learning in the original manuscript have caused certain confusion. We have changed the title to “**Hybrid computational modeling demonstrates the utility of simulating complex cellular networks in type 1 diabetes**”. We have also addressed the reviewers’ comments point-by-point. Please see our response as follows.

**Reviewer Comments:**

**Reviewer #1:**

Shi and co-authors present a study aimed at integrating data-driven and mechanistic (agent-based) modeling in the context of the immune dysregulation underlying Type 1 Diabetes (T1D). Overall, I am concerned that the authors conflate the data-driven and statistical methods used in the analysis of model output with a true integration of data-driven and mechanistic modeling. I would argue that to truly integrate these modeling approaches, the authors would have had to use machine learning approaches to help define model rules and/or parameters, rather than simply using those tools to analyze model output. I am also concerned that no true, prospective validation of key model predictions has been carried out. Thus, it is unclear to me that such a nominally complex architecture was needed in order to perform the studies presented in the manuscript.
**Response:**  We would like to thank Reviewer #1 for the time and effort put into the evaluation of our manuscript. To clarify the concept of our modeling approach and answer some concerns from Reviewer #1, we would like to review our modeling approach from a top level and then provide a point-by-point response as follow.

Computational systems biology, especially modeling disease progression through a complex intra- and inter-cellular network is a challenging area. The key challenges, among other things, include integrating detailed biology of immune responses into a systematic framework, estimating a large number of unknown biological parameters, and validating the modeling framework based on known mechanisms and/or experimental data.

Starting from 2016, we have worked on developing a hybrid computational framework to stimulate complex intra-cellular networks during type 1 diabetes mellitus (T1DM) progression. This idea has emerged since I started to work on mathematical modeling of T1D several years ago. At that time, we focused on predictive models using systems of integro-differential equation to define sets of criteria that determined susceptibility and autoimmune responses in T1DM using quantitative methods to understand onset, progression, and prevention of the disease. However, these approaches are highly limited to integrating and defining multifaceted pancreatic cellular interactions, and highly unlikely and impractical to describe population heterogeneity and intrinsically orchestrated and noise-driven processes of complex biological systems such as T1D.

This concern was also raised by experts in the field of immunological tolerance and autoimmune diseases including Dr. Christophe Benoist, Professor of Microbiology and Immunobiology at Harvard Medical School. In our communication, Dr. Benoist mentioned that traditional multi-compartment ODE modeling cannot well capture their experimental data regarding the dynamics of infiltrating CD8+ T cells in NOD mouse models. For this purpose, I have recruited Dr. Zhenzhen Shi as a postdoc (now she is a Research Assistant Professor at Loyola University Chicago Medical School), who is an expert in agent-based modeling of complex biological systems, in our Institute of Computational Comparative Medicine. We all agreed that agent-based modeling (ABM) can model cell heterogeneity from single-cell variations to mixed cells which then can reflect the noise-induced cellular interactions in a systematic way and eventually predict individual-specific behaviors of the complex biological system.

By reviewing existing ABMs in the literature, we realized that the rigorous statistical analysis of simulation data should be conducted to improve the predictability of ABM, which is not included or well performed in most existing ABMs. A review paper recently published:

1. [Williams, Richard A. "Lessons learned on development and application of agent-based models of complex dynamical systems." Simulation Modelling Practice and Theory 83 (2018): 201-212.](https://www.sciencedirect.com/science/article/abs/pii/S1569190X17301557)

The paper pointed out the importance of thorough verification and rigorous analysis of data simulation during ABM development.

Thus, we have developed a hybrid computational framework by incorporating biological parameters, mathematical components, and statistical methods into our ABM to unravel the aforementioned challenges. To provide a historical overview of the work, it is worth mentioning that it took us almost 4 years to start from the design of an initial model to the development of this hybrid computational framework. The proposed computational framework which is applied to simulate T1DM progression has linked agent-based modeling, mathematical modeling, and parameter estimation to a detailed description of noise-driven processes of complex biological systems.

It is also worthwhile to point out that the significance of our discovery in the study of T1D progression through computation has been validated and enriched by experimental data and experimental findings. Model results were validated by experimental raw data provided by Dr. Christophe Benoist from Harvard Medical School and further validated by abundant experimental findings in the literature. We discovered essential biological parameters that are significantly correlated to outcomes of T1D progression. These results by employing local and global sensitivity analyses encouraged us to conduct therapy-directed simulations as potential targets for treatment strategies that have not been investigated experimentally to prevent overt T1D.

It is also worth mentioning that we agree that using the words “connecting data science and statistical learning” in our title could be misleading and, for this reason, the title was revised to reflect better our work. The local sensitivity and global sensitive analysis (part of statistical learning) were used to analyze the outputs of this hybrid computational framework rather than build the framework.

1. Introduction: Some of the statements made by the authors are paradoxical. For example, they state that “…the barrier in sampling experimental data from pancreatic tissues or lymph nodes in high-risk T1D subjects makes the use of quantitative modeling approaches based on AI machine learning of pancreatic β-cell destruction an intriguing opportunity to analyze this disease.” It would seem that the exact opposite is true: the absence of extensive time course data would appear to make data-driven modeling/machine learning very difficult. They also refer to “mathematical data science models” and “data-oriented agent-based modeling,” which to me is an oxymoron: mechanistic mathematical and agent-based models are based on biological principles derived from the aggregate knowledge gleaned from the literature, rather than on specific datasets (though, of course, mechanistic models can be calibrated to data). In contrast, data-driven/machine learning models are largely based on data rather than on principles. The authors need to read through the paper carefully and make sure that such statements, which make it appear as if the authors are not familiar with these core concepts, are modified or removed.

**Response:**  Thanks for this great comment. To be trouthful, we plan to combine the results of this studywith our recently published papers based on pharmacovigilance and adverse drug events using AI and data driven methods in the near future. However, in this manuscript, we should have only highlighted model outputs that were analyzed using data-oriented methods including local and global sensitivity analysis. We realize that using “data-oriented or AI machine learning” in the manuscript would be misleading. In this revision, we have removed those terminology to avoid any confusion.

2. Introduction: the authors mostly cite their own prior work on modeling T1D. Even a cursory search of PubMed lists a substantial number of mechanistic models of T1D in addition to the one paper cited by Linderman et al, and these studies should be acknowledged and discussed. Likewise, there have been review/perspective articles that have called for the integration of mechanistic and data-driven modeling approaches, and some of those papers should be cited as well.

**Response:**  Thanks for your comment. We added the following paragraph to discuss other relevant literature.

“Linderman et al. applied an agent-based approach to examine the relationships between parameters related to dendritic cell-T cell interactions and the output of primed T cells (19). There are also a number of agent-based models focusing on different aspects of T1D progression. Wedgwood et al. developed an agent-based model to investigate the progression of insulitis in human T1D by simulating interactions among B cells, T cells, and beta cells (20). Additionally, Ozturk et al. proposed an agent-based model focusing on modeling interaction between beta cells and different types of CD8^+^ T cells (21). Later, Xu et al. proposed another agent-based model to simulate the effect of regulatory T cells on T1D progression (22). Compared to the previous studies, we focus on modeling the detailed biology of dendritic cells and their interactions with CD8^+^ T cells, beta cells, and antigens. Moreover, thorough and expensive statistical methods were utilized to analyze the outputs of the model and provided a solid foundation for therapy-directed simulations. This model was also designed to investigate individual-specific dynamics of CD8^+^ T cells which were observed in NOD mouse experiments.”

3. Introduction: the paragraph which introduces the NOD mouse model is jumbled and should be revised. As written, it is difficult to tell if the authors are saying that obtaining data in this model is useful or not, as they appear to argue against themselves.

**Response:**  Thanks for your comment. We have revised the paragraph to make a clear statement that obtaining data in NOD mouse models is useful for three reasons: (1) identifying safe methods for sampling and visualizing the human endocrine pancreas remains incompletely understood; (2) the NOD mouse models share a number of genetic and immunologic traits with human T1D which serves as prominent tools for studying the pathogenesis of T1D in humans; and (3) an abundance of data is accessible for accurate incorporation of relationships among agents to support the establishment of ABM. Please see the revised paragraph as follows:

“The non-obese diabetic (NOD) mouse is a well-established animal model that exhibits striking similarities to the disease in humans. NOD mouse models sharing a number of genetic and immunologic traits with human T1D (23-25) serve as testing arenas for potential treatments and reliable and prominent tools for studying the pathogenesis of T1D in humans (26). Since identifying safe methods for sampling and visualizing the human endocrine pancreas remains incompletely understood (27), autoimmunity during T1D progression has been investigated extensively in NOD mouse models (23, 24). Therefore, an abundance of data is accessible for accurate incorporation of relationships among agents to support the establishment of ABM.”

4. Introduction (line 123): the authors do not give an adequate rationale as to why a hybrid model is the best approach. As mentioned in point #1 above, the authors argue that data are insufficient, and in point #3 they make a weak case for obtaining data in the NOD mouse model. Based on that, I do not see how “…based on the above facts, (they) proposed a novel hybrid agent-based model by integrating phase progressions in complex pancreatic cellular networks, mathematical modeling, biological parameters in the domain of machine learning models, ABM, and statistical learning components into a computational framework to simulate T1D progression using experimental data and clinical evidence from NOD mouse models.” I would argue that the exact opposite is true, based on those same facts. Ultimately, the authors have chosen to take this approach, but the “facts” as given are not sufficient support for why they chose to do so.

**Response:** As mentioned above, “data-driven ABM” and other AI-related keywords in the original manuscript have caused noticeable confusion and, therefore, deleted. The core of this hybrid model is to simulate T1D progression using biological principles rather than on specific datasets. But thanks to the raw data and other experimental pieces of evidence obtained from NOD mouse models, we are able to validate the model and use the model to identify non-intuitive parameters contributing to the T1D progression.

As we stated on pages 3-5 of this response letter as well as on pages 4-6 of the original manuscript, one motivation of building such a framework is because that our previous models using systems of integro-differential equation are highly limited to integrating and defining multifaceted pancreatic cellular interactions, and highly unlikely and impractical to describe population heterogeneity which has been identified in experimental findings.

The other important aspect of this study is to conduct rigorous statistical analysis for the ABM outputs. In the existing ABM approach for complex disease progression including T1D progression, thorough statistical analysis has not been conducted or well performed. As also raised by Reviewer #2, we have included additional literature to make it clear. As such, in the Introduction we have modified the original paragraphs and added a new paragraph to explain why this hybrid model structure was chosen:

“The barrier in sampling experimental data from pancreatic tissues or lymph nodes in high-risk T1D subjects makes the use of mathematical/computational modeling of pancreatic β-cell destruction an intriguing opportunity to analyze this disease. Various questions and problems typically arise from researchers and the disease on a daily basis that require long and tedious experimental work to possibly find an answer. Since the model has been trained on multiple datasets, having such a practical and flexible platform can widen the horizon with reasonable accuracy and provide preliminary insight into the appeal of such questions as the ABM model can be quickly adjusted to address such inquires and issues before conducting any experiments. In addition, we can all acknowledge designing an experiment requires lengthy paperwork and follows with associated costs assuming all the tools, materials, patients are available for lab work. Moreover, experiments alone cannot often explain the behavior of very rich and complex developmental dynamics of pancreatic islets and β-cells with feedback across different levels of biological organization as pointed out in Anmar and Santiago’s review (1). Such a quantitative approach emerging from modeling the detailed biology of immune responses could provide novel insights into the mechanisms underlying the regulation of T1D when the safety, reproducibility, and efficiency of the present experimental techniques are challenging.”

“Now the challenge is to design and construct such a quantitative framework for analyzing highly orchestrated biology, immunology, and the pathogenesis of T1D based on the current knowledge and modular organization of cellular networks and biological parameters. To unravel complex system behaviors resulting from the inter- and intra-cellular and signaling networks linking the immune system and metabolism during T1D progression, it is crucial to first integrate essential information from inherent biological processes into a systematic framework.”

“Mathematical models have been widely employed to study systematic behaviors in disease progression (2-8). Our previous studies focused on the mathematical modeling of T1D progression using novel object-oriented approaches such as integro-differential equation modeling with reasonably defined sets of criteria that determined susceptibility to T1D (9-12). Compared to mathematical models, agent-based modeling (ABM) has been used to model cell heterogeneity from single-cell variations to mixed cells by simulating the behaviors of individual agents (13-17). Depending on the behaviors of individual agents in a biological system, agents interact with each other and move over a simulation environment following a set of pre-defined rules (18).”

“Linderman et al. applied an agent-based approach to examine the relationships between parameters related to dendritic cell-T cell interactions and the output of primed T cells (19). There are also a number of agent-based models focusing on different aspects of T1D progression. Wedgwood et al. developed an agent-based model to investigate the progression of insulitis in human T1D by simulating interactions among B cells, T cells, and beta cells (20). Additionally, Ozturk et al. proposed an agent-based model focusing on modeling interaction between beta cells and different types of CD8+ T cells (21). Later, Xu et al. proposed another agent-based model to simulate the effect of regulatory T cells on T1D progression (22). Compared to the previous studies, we focus on modeling the detailed biology of dendritic cells and their interactions with CD8+ T cells, beta cells, and antigens. Moreover, thorough and expensive statistical methods were utilized to analyze the outputs of the model and provided a solid foundation for therapy-directed simulations. This model was also designed to investigate individual-specific dynamics of CD8+ T cells which were observed in NOD mouse experiments.”

“The non-obese diabetic (NOD) mouse is a well-established animal model that exhibits striking similarities to the disease in humans. NOD mouse models sharing a number of genetic and immunologic traits with human T1D (23-25) serve as testing arenas for potential treatments and reliable and prominent tools for studying the pathogenesis of T1D in humans (26). Since identifying safe methods for sampling and visualizing the human endocrine pancreas remains incompletely understood (27), autoimmunity during T1D progression has been investigated extensively in NOD mouse models (23, 24). Therefore, an abundance of data is accessible for accurate incorporation of relationships among agents to support the establishment of ABM.”

“Based on the above facts, we proposed a novel hybrid agent-based model by integrating phase progressions in complex pancreatic cellular networks, mathematical modeling, biological parameters, and ABM into a computational framework to simulate T1D progression using experimental data and clinical evidence from NOD mouse models. Through exploiting ABM, the aim of this study includes the following four aspects: (1) to develop a hybrid model based upon the underlying mechanisms summarized from the NOD mouse model and then cross-validate the model with experimental data; (2) to predict pancreatic β cell destruction targeted by the immune system during T1D progression; (3) to identify key parameters in driving autoimmunity and estimate effects of these parameters on determining how sensitive (or uncertain) different mechanisms of the model are to perturbations and thus identifying potential targets for therapeutic purposes; and (4) to conduct therapy-directed simulations and explore immune-intervention strategies for predicting the onset and progression of the disease and potentially treating T1D.”

5. Results: the authors jump right to validation of their model against data. They need to first describe the model at least in high level (abstracting what is in the Methods) and give key performance metrics of the model (e.g. ability to simulate important divergent outcomes associated with progression, or lack thereof, to T1D). This brings up another important point: are the authors simulating a state of existing T1D, or a progression to T1D (or not, depending on simulation conditions) that then can be queried and perturbed? The latter would be preferable to the former, because simulating a situation where the investigators simply posit that “this is T1D” means that they have fully addressed the dynamics of the biological processes that lead up to that point. The statements further down suggest that the model is in fact one of T1D progression, but this needs to be made explicit and supported with details of the model. Furthermore, it is unclear what role machine learning played in the generation of this model. Was a data-driven modeling analysis performed in order to help define model rules, key interactions, feedbacks, or was machine learning only used in the analysis of the parameterization and output of the model? If the latter, then this is really not novel as this type of analysis of output of mechanistic models has now become fairly standard.

**Response:**  Thanks for your comments. Due to the limitation, we needed to give a summary of the important findings that’s why we had to move right to the validation section. In this revision, we present the Materials and Methods section earlier (before the Results section) in the main text to clarify the model concept. In addition, we moved the mathematical model section, experimental data collection section, parameter estimation section, local sensitivity analysis section, global sensitivity analysis section, and therapy-directed simulation section from Supplementary Materials to the main text to further clarify modeling details. A high-level abstract describing key performance metrics of the model was added to the Methods section as follows:

“**Abstraction of modeling performance**: This hybrid computational modeling is designed to simulate a T1D progression, i.e., starting with the detection of antigens in pancreatic islets to possible outcomes of overt T1D. We focus on modeling interactions between a class of agents including antigen, pancreatic β cell, dendritic cell, naive CD8+ T cell, activated CD8+ T cell, and cytotoxic CD8+ T cell. The rules that govern interactions between these agent classes were summarized based on existing literature (which is described in detail in the Agent behaviors section). The selection of an agent class was based on expert knowledge and available experimental data of infiltrating CD8+ T cells. Under different biological conditions, the model can predict the individual-specific trajectory of infiltrating CD8+ T cells. The model can also predict the time interval between antigen detection to the possible development of overt diabetes to mimic the differentiation in T1D progression. Moreover, the model provides a computational platform to test the efficacy of different therapeutic strategies.”

As stated in the abstraction above, the model was designed to simulate a T1D progression not only a state of existing T1D. Depending on different conditions, a T1D progression can develop into overt T1D and through the thorough statistical analysis, those conditions can be identified by the model (as shown in **Fig. 6** and **Fig.7**).

The main role of statistical learning is to analyze the model outputs but as we stated earlier in this response letter, the novelty of this study is to construct such a complex modeling framework for simulating T1D progression using a hybrid of agent-based and mathematical modeling with experimental data validation, substantial statistical analysis, and therapy-directed simulation which have not been fully addressed in the existing modeling study for T1D progression.

6. Results, Fig. 1: what was the number of experimental repeats (animals/samples)? Also, the authors state that “…a significant increase in infiltrating CD8+ T cells occurs around week 8 in the simulation.” How do they define “significant”? Was a statistical analysis carried out? Also, I am concerned that the relatively tight confidence intervals in the data have resulted in an overfit model (see my point #7 below).

**Response:**  The number of experimental repeats was illustrated in the legend of **Fig.4**: Infiltrating CD8+ T cell counts were illustrated using the raw data (7 replications at week 4 and week 12, 6 replications at week 6 and week 10, 5 replications at week 8, and 12 replications at week 14). A significant increase (~7% from the baseline) around week 8 was observed using a Chi-squared test, that is where massive assault to beta cells occurs. Also it is worth mentioning that the figures are not showing very tight moving error-bars since we are using the standard error of the mean (SEM) as opposed to a typical standard deviation (SD) that show much higher fluctuations.

7. How does the Latin Hypercube Sampling approach to defining parameter values handle the effect of stochasticity in the ABM? How were these parameter choices validated? How is overfitting avoided? In the clinical setting of T1D, I would expect much larger confidence intervals around the dynamics of cells and inflammatory mediators, and hence parameter choices that appear to work in this experimental setting may not be useful in truly understanding T1D.

**Response:**  Latin hypercube sampling (LHS) is a technique of sampling that can be used to produce input values for the estimation of expectations for the output variables. LHS is more efficient than simple random sampling in a large range of conditions and can help with the uncertainty and sensitivity analysis by providing various applications and performance including: (i) stratifying and variance decomposition, (ii) involving correlation control, reweighting of samples to incorporate changed distributions, or replicating sampling to test the reproducibility of results, and (iii) and helping in performing analyses involving stochastic or subjective uncertainty. LHS uses a stratified sampling scheme (*n=*100) to improve on the coverage of the *m-*dimensional input space of feasible model parameters (*m=*5). This means that a single sample drawn from the m-dimensional space of parameters will provide useful information when some input variable(s) dominate certain responses (or certain time intervals). By sampling over the entire plausible range, each variable (cell) has the opportunity to show up as important, if it indeed is important. The values of the stratified sampling scheme can be paired to ensure a desired correlation structure among the n input variables. In our analysis, the asymptotic variance of an estimate is obtained. The LHS approach was conducted ten times for each selected parameter combination to capture the stochasticity in the ABM. Please see the parameter estimation section in the manuscript:

“**Parameter estimation:** The Latin hypercube sampling (LHS) method is applied to estimate the default values of unknown parameters. The ranges of unknown parameters were summarized from literature and obtained from the field experts’ suggestions, which are included in **Table S4** (i.e. Ranges used for LHS). Based on the LHS method, the range of each parameter value is divided into n = 100 intervals and each interval of a parameter is sampled once. A total of 100 combinations of parameters generated from the LHS Package in R was performed on our local server to obtain the dynamics of CD8^+^ T cells and compare them with the experimental data. About 25 simulations were run simultaneously each time on the server (server-specific configuration: 4x Intel Xeon CPU E5-4650 2.1 GHz 48 cores with 192 GB of RAM). In addition, each set of parameters was then performed 20 times to obtain the average counts of infiltrating CD8+ T cells at week 4, week 6, week 8, week 10, week 12, and week 14. A set of parameter values was selected as the default values that satisfy the condition where the difference between the simulated results and experimental data at weeks 4, 6, 8, 10, 12, and 14 is optimally minimized. The results then showed that kinetics of CD8^+^ T cells highly follow oscillatory dynamics as observed and suggested by studies and experts (56-58). Parameter ranges utilized for local sensitivity analysis (LSA) are included in **Table S4**. The ranges determined by the field experts are marked using asterisks. For other ranges, appropriate references are provided in **Table S4**.”

The selection of parameter was based on the selection of agents and their interactions summarized from the existing literature (agent behaviors section)

There is no concern about overfitting as well, on the contrary, we ran the model on the entire ranges of parameters (that were summarized from literature and obtained from the field experts’ suggestions) and used population heterogeneity of all the agents following their own intrinsically orchestrated and noise-driven processes of complex biological systems. It did actually cause huge variations instead of causing to fit perectly. Tight CI is because we are showing SEM and not SD.

We agree that there is a discrepancy between the NOD mouse model and the human model. We have compared such discrepancy in the Disucssion section:

“A second limitation concerns the discrepancy between the NOD mouse model and the human model. Despite the NOD mouse model has been successful in multiple forms for studying T1D in humans (23), the limitation of the NOD mouse model should be marked. Compared to the NOD mouse model, it seems that the human pancreas has a lower potential for β-cell regeneration based on recent access to the pancreas of organ donors with T1D (103). In addition, the severity of insulitis in human models has been found to be less pronounced than in the NOD mice model (104), which may impact the conclusions that parameters associated with CD8^+^ T cells predominate T1D progression in human models. Based on these reasons, it is worth mentioning that the strategies that would be effective in the NOD mouse model are not necessarily effective in clinical trials. We also considered modeling other treatment parameters such as anti-CD3 because they were proven to be effective in NOD mice and clinical trials (105).”

8. Results, Fig. 2: As per point #6, the authors again state that “…counts of healthy β cells start to

9. significantly decrease at around week 8.” Again, how was significance determined?

**Response:**  Yes, there has been a massive assulat from T-Cells and it is significant. As discussed previously, the significant decline can be easily show by a Chi-squared test around week 8 where a massive assault to beta cells occurs.

10. Results: The authors state that “…(they) applied sensitivity analysis methods to investigate the main and interaction effects of unknown parameters on T1D development, and their domain of machine learning.” What does “their domain of machine learning” mean? This is a key point, since I am still unclear as to what role machine learning has played in these analyses (see my point #5 above).

**Response:**  We agree that “their domain of machine learning” should be removed to avoid any confusion.

11. Figs. 5 & 6: no details are given as to what “statistical learning” was carried out (and why).

**Response:**  We have removed “statistical learning” to avoid confusion.

**Reviewer #2:**

The manuscript reports the development of a hybrid computational modeling based on agent-based-modeling (ABM) to simulate some aspects of the complex cellular networks in type 1 diabetes (T1D). A comprehensive effort is made to investigate the development of Type 1 diabetes based on the behavior of some components of the immune system. The model is validated by data from NOD mice. The contributions and clarity of the manuscript can be enhanced by addressing the issues listed below.

**Response:**  We would like to express our gratitude towards Reviewer #2 for the time and effort put into the evaluation of the manuscript. Please see our point-by-point response as follows.

The structure of the agent-based model (the structure of the agents, the layers, Net-Logo) should be presented earlier in the text to inform the readers about the characteristics and limitations of the ABM developed. One of the agents should also be given to illustrate how the agents developed are interacting and the level of “intelligence” of the agents – how they perceive their environment, how the decide on their action, and how these actions are implemented.

**Response:**  Thanks for your comment. We moved the Materials and Methods section above, so it now appears before the Results section. We also moved other Materials and Methods parts from the Supplementary Materials to the main text to clarify the development of ABM. In the original paper, we use **Fig. 8** to illustrate all the interactions between multiple agents. But followoing the reviewer suggestion to give a better illustration of the level of “intelligence”, we have included the following diagram (**Fig. 3**) to show how the agents have been developed and interacted with others in more detail.

The components of the immune system included in the model and the components left out that may have an effect on the progress should be described clearly. All interactions of various activities of the immune system that lead to T1D may be too complex to fit in a Net-Logo model or even to develop their rules. But it would be valuable to the readers to know the limitations of the model and the phenomena that it describes/simulates.

**Response:**  Thanks for your comment. We have explained the reasons why some components of the immune system were excluded. Please see the following texts in the Discussion part:

“The present study should be evaluated in the context of several possible limitations. First and foremost, like all models, the hybrid computational model presented herein is an abstract of reality, to some extent, similar to an experiment when it is performed for a laboratory investigation dealing with a controlled environment while it might give a completely different output in reality and human body. Although the model was designed to describe the major components based on the current knowledge and available data, excluded multiple mechanisms associated with T1D progression should be mentioned herein. For example, we did not model CD4^+^ T cells in this study because the role of CD4^+^ T cells in autoimmune T1D remains incompletely understood (35) and thus experimental data on CD4^+^ T cells are not sufficient. It is worthwhile to mention that the progression of T1D is associated with the finely tuned immune balance between effector CD4^+^ T cells and regulatory CD4^+^ T cells. The quantitative relationship between regulatory CD4^+^ T cells and effector CD4^+^ T cells is regulated by other cell types and cytokines (32, 35, 99-101). In this case, additional interactions between dendritic cells, CD4^+^ T cells, and CD8^+^ T cells would require extensive efforts in estimating unknown parameters and their distributions. When the number of unknown parameters is very large, it is often impossible to identify reliable model results (102)”

An exploratory sensitivity analysis is made by varying one input at a time. Of course, this does not permit capturing the effects of interactions between the inputs that may have significant effects on the outputs. A cautionary note to warn the reader would be useful.

**Response:**  Yes, we applied local sensitivity analysis to investigate the effect of variying one input at a time on the model output. Then, the effect of interactions from multiple inputs on the model output was explored by using global sensitivity analysis. We have added additional texts in the local sensitivity analysis section to clarify the flow of our manuscript:

“Within the sensitivity analysis, local sensitivity analysis quantifies the effect of small variations of input factors on output variables when only one factor is changed at a time (59). A global sensitivity analysis was conducted later to investigate the effect of multiple interactions between the inputs and model output. Compared to the global sensitivity analysis (as described in the next section), local sensitivity analysis requires less computational costs (60)”

Fourier amplitude sensitivity test (eFAST) method is used for global sensitivity analysis. Some brief remarks on why this method is preferred over Sobol sensitivity analysis should be included in the text.

**Response:**  Thanks for your comment. We have added the following paragraph to address this comment and justify why we choose eFAST over Sobol’s method:

“**Global sensitivity analysis:** Global sensitivity analysis is a technique applied to simulations of the performance and the quantitative effect of input variables on output variables when the input variables are varied over the entire possible ranges (60). Botj extended Fourier amplitude sensitivity test (eFast) and Sobol’s method deliver the measures for the decomposition of the total variance of model output into the main and interaction effect of each parameter. Compared to Sobol’s method, eFast is more computationally efficient because it requires fewer sampling/simulations (60). As such, we implemented an eFast, which is an advanced version of the Fourier amplitude sensitivity test (FAST).”

The destruction of islets and beta cells by using ABM was reported by Xu, Ozturk, and Cinar in some recent papers: Agent-Based Modeling of Immune Response to Study the Effects of Regulatory T Cells in Type 1 Diabetes. Processes, 2018; and Agent-Based Modeling of the interaction between CD8+ T cells and Beta cells in Type 1 Diabetes, PLoS ONE, 2018. Their ABM is also based on mouse models. These should be included in the references and some of their results should be compared with the results of the ABM model reported in this manuscript.

**Response:**  Additional reference has been included in the manuscript. Please see the following paragraph:

“Linderman et al. applied an agent-based approach to examine the relationships between parameters related to dendritic cell-T cell interactions and the output of primed T cells (19). There are also a number of agent-based models focusing on different aspects of T1D progression. Wedgwood et al. developed an agent-based model to investigate the progression of insulitis in human T1D by simulating interactions among B cells, T cells, and beta cells (20). Additionally, Ozturk et al. proposed an agent-based model focusing on modeling interaction between beta cells and different types of CD8^+^ T cells (21). Later, Xu et al. proposed another agent-based model to simulate the effect of regulatory T cells on T1D progression (22). Compared to the previous studies, we focus on modeling the detailed biology of dendritic cells and their interactions with CD8^+^ T cells, beta cells, and antigens. Moreover, thorough and expensive statistical methods were utilized to analyze the outputs of the model and provided a solid foundation for therapy-directed simulations. This model was also designed to investigate individual-specific dynamics of CD8^+^ T cells which were observed in NOD mouse experiments.”

The terms AI, machine learning, and AI machine learning are used in the manuscript several times. The ABM is a system with distributed AI, and this should be mentioned when the ABM is introduced. The use of machine learning should be pointed out and the term “AI machine learning“ should be clarified or removed. Machine learning encompasses the capture of underlying relations or behavior in data by processing the data. Most of the results reported are the results of simulations. The results are reported by using statistical analysis techniques such as ANOVA. If there are any automated classifications (for example by using support vector machines, neural networks, discriminant analysis) the term machine learning would be appropriate and pointed out in the manuscript.

**Response:**  Thanks for pointing this out. We have removed “AI machine learning” and other relevant keywords to avoid any confusion.
